# Non-adherence to the single dose nevirapine regimen for the prevention of mother-to-child transmission of HIV in Bindura town, Zimbabwe: a cross-sectional analytic study

**DOI:** 10.1186/1471-2458-10-218

**Published:** 2010-04-28

**Authors:** Lazarus R Kuonza, Clemence D Tshuma, Gerald N Shambira, Mufuta Tshimanga

**Affiliations:** 1 Department of Community Medicine, College of Health sciences, University of Zimbabwe, Harare, Zimbabwe; 2 Ministry of Health and Child Welfare, Mashonaland Central Province, Bindura, Zimbabwe

## Abstract

**Background:**

The Prevention of Mother to Child Transmission of HIV (PMTCT) programme was introduced at Bindura Hospital in 2003. Seven additional satellite PMTCT clinics were set up in the district to increase service coverage but uptake of PMTCT interventions remained unsatisfactory. In this study we determined the prevalence of and factors associated with non-adherence to the single dose nevirapine (SD-NVP) regimen for PMTCT in Bindura town.

**Methods:**

An analytic cross-sectional study was conducted in four health institutions in Bindura town. Participants were mother-baby pairs on the PMTCT programme attending routine six weeks post natal visits in the participating health institutions from March to July 2008. We interviewed 212 mothers using a structured questionnaire.

**Results:**

The non-adherence rate to the maternal nevirapine dose was 30.7%, while non-adherence to the newborn nevirapine dose was 26.9%. The combined mother-baby pair nevirapine non-adherence was 42.9%. Non-adherence to the maternal dose of nevirapine was associated with lack of maternal secondary education (POR = 2.38; 95%CI: 1.05-3.39) and multi-parity (POR = 2.66; 95%CI: 1.05-6.72), while previous maternal exposure to the PMTCT programme (POR = 0.22; 95%CI: 0.08-0.57) and giving the mother a NVP tablet to take home during antenatal care (POR = 0.03; 95%CI: 0.01-0.09) were associated with improved maternal adherence to nevirapine. Non-adherence to the infant dose of nevirapine was associated with maternal non-disclosure of HIV results to sexual partner (POR = 2.75; 95%CI: 1.04-7.32) and home deliveries (POR = 48.76; 95%CI: 17.51-135.82).

**Conclusions:**

Non-adherence to nevirapine prophylaxis for PMTCT was high in Bindura. Ensuring institutional deliveries, encouraging self-disclosure of HIV results by the mothers to their partners and giving HIV positive mothers nevirapine doses to take home early in pregnancy all play significant roles in improving adherence to PMTCT prophylaxis.

## Background

Zimbabwe has the 4^th ^highest rate of HIV infection in the world, with an estimated HIV prevalence of 15.6% in adults aged 15-49 years [1,2]. The HIV pandemic has increasingly affected children, resulting in the reversal of the gains that had been made in the reduction of infant and childhood morbidity and mortality in the country.

Mother-to-child transmission of HIV (MTCT) during pregnancy, delivery, or breastfeeding accounts for more than 90% of the HIV infections in children below 15 years of age [3,4]. Without interventions, 20-45% of babies born to HIV positive women become vertically infected with HIV [5]. Studies have shown that short-course, easy-to-use, and affordable antiretroviral regimens can significantly reduce MTCT of HIV. One such regimen is the single-dose nevirapine (SD-NVP) regimen, in which the mother ingests a dose of nevirapine (NVP) at the onset of labor (at least 2 hrs before delivery) and another dose is given to the baby within 72 hrs of birth [4,6-8].

In Bindura district the PMTCT programme was started in 2003 at Bindura Hospital. In 2004 seven additional satellite comprehensive PMTCT clinics were set up in the district to increase the coverage of the services. Despite these efforts, the uptake of the PMTCT interventions remained unsatisfactory. During the period January to May 2007 1161 mothers delivered at Bindura Hospital. Of all these mothers, only 248 (21%) were tested for HIV before giving birth, the rest (913) had unknown HIV status and thus could not benefit from the PMTCT programme. Out of the 248 mothers who were tested for HIV before delivery, 65 (26.2%) were HIV positive. Of these 65, only 43 (66.2%) swallowed the recommended nevirapine doses before giving birth [9].

This then raised questions on why some pregnant mothers took the bold step of being tested for HIV but would not complete the recommended protocols for them to prevent the transmission of HIV to their babies.

In Zimbabwe the SD-NVP regimen remains the recommended prophylaxis for PMTCT. The government is planning to adopt more efficacious regimens, but the problems affecting the current SD-NVP regimen with regard to access, uptake and adherence, may still affect these future regimens. Identifying these problems can assist in planning the rolling out of the new PMTCT regimens. In this study we therefore set out to determine the extent of non-adherence and to identify factors associated with the non-adherence to the SD-NVP regimen in Bindura.

## Methods

An analytic cross-sectional study was conducted in the four health facilities that provide comprehensive PMTCT and maternity services in Bindura town. The study population included mothers-baby pairs registered on the PMTCT programme and attended routine six-week postnatal care in these health facilities. We reviewed the health facility PMTCT registers and all registered HIV positive mothers (and their babies) who visited these heath facilities for postnatal care between March and July 2008 were enrolled into the study. We excluded mothers who were on antiretroviral therapy before giving birth. The minimum sample size was calculated as 197 mothers-baby pairs, using the formulae: [10], assuming an error risk (z_α_) of 1.96, with a precision (Δ) of ± 5% and expecting that 15.1% (*p*) of the mothers would be non-adherent (*as reported by Albreicht et al in the study: Predictors of non-adherence to single dose nevirapine for PMTCT in Lusaka Zambia*) [11].

The mothers were interviewed using a structured questionnaire to collect information on socio-demographic, socio-economic, socio-cultural variables, knowledge on PMTCT as well as perceptions on health services being provided.

Ethical approval to conduct the study was granted by institutional review boards of the Provincial Medical Directorate (PMD) for Mashonaland Central Province, the Bindura Municipality Directorate of Health Services and the Health Studies Office of the Ministry of Health. The aim of the study was explained to all the potential participants and written consent was obtained before the interviews. Confidentiality was assured and maintained throughout the study and no identifying information was collected.

Statistical analyses were conducted using the Epi-info statistical software (CDC-Atlanta, 2007) [12]. We first performed descriptive analyses, looking at the extent of non-adherence to NVP and comparing the distribution of various characteristics among the non-adherent and adherent mother-baby-pairs. Non-adherence was defined as lack of ingestion or delayed ingestion of NVP by either the mother or the baby (delayed ingestion being taken as less than 2 hours before delivery for the mother and more than 72 hours after birth for the baby). We then performed bivariate analyses of the possible associations between non-adherence and the variables under investigation. In determining these associations prevalence odds ratios (PORs) and their 95% confidence intervals (95%CI) were calculated. We then conducted stratified analysis to assess for possible effect modification (interaction) and confounding. Lastly we performed logistic regression analyses where all independent variables that were significant at the 0.25 level (χ^2^, *p *≥ 0.25) in bivariate analyses were included in the model [13], to come up with adjusted PORs and 95%CIs.

## Results

We interviewed 212 mothers between March and July 2008. Of the 273 PMTCT mothers who were due to attend the six-week postnatal care in the participating health institutions during the study period, 229 (83.9%) attended the postpartum visit. Seventeen mothers were excluded for failing to meet the inclusion criteria, 14 had been on antiretroviral therapy before giving birth while the other three indicated that they were unwilling to respond to questions. The majority of the participating mothers, 131 (61.8%), were aged between 21 and 30 years, with a median age of 27.0 yrs (Q1 = 23; Q3 = 32). The mothers were largely married (formally or informally), 170 (80.2%) and most, 150 (70.8%), had attained at least secondary education. Of the 212 mothers only 63 (29.7%) were gainfully employed (Table 1).

**Table 1:** Demographic characteristics of the PMTCT mothers who participated in the study, Bindura, 2008

Characteristic	Total		Non-Adherent		Adherent		p-value
	n = 212	%	n = 91	(%)	N = 121	(%)	
**Place of residence**							
Urban	**114**	***(53.8)***	29	*(31.9)*	85	*(70.2)*	<0.001
Commercial Farming areas	**41**	***(19.3)***	27	*(29.7)*	14	*(11.60*	<0.001
Peri-urban	**27**	***(12.7)***	15	*(16.5)*	12	*(9.9)*	0.15
Communal (rural)	**23**	***(10.8)***	14	*(15.4)*	9	*(7.4)*	0.06
Mining community	**7**	***(3.3)***	6	*(6.6)*	1	*(0.8)*	0.05
							
**Marital status**							
Married/co-habiting	**170**	***(80.2)***	70	*(76.9)*	100	*(82.6)*	0.30
Single	**16**	***(7.5)***	9	*(7.4)*	7	*(7.7)*	0.26
Divorced/separated	**15**	***(7.1)***	10	*(11.0)*	5	*(4.1)*	0.09
Widowed	**11**	***(5.2)***	4	*(4.4)*	7	*(5.8)*	0.76
							
**Highest level of education attained**							
Up-to primary	**62**	***(29.2)***	42	*(46.2)*	20	*(16.6)*	<0.001
Secondary (up-to form 4)	**138**	***(65.1)***	46	*(50.6)*	92	*(76.0)*	<0.001
Secondary (Form 5 and 6)	**10**	***(4.7)***	3	*(3.3)*	7	*(5.8)*	0.40
Tertiary	**2**	***(0.9)***	0	*(0)*	2	*(1.7)*	0.22
							
**Employment status**							
Formally employed	**26**	***(12.3)***	4	*(4.4)*	22	*(18.2)*	0.002
Informally employed	**37**	***(17.5)***	18	*(19.8)*	19	*(15.7)*	0.44
Not employed	**149**	***(70.3)***	69	*(75.8)*	80	*(66.1)*	0.13

### Maternal adherence to nevirapine

Out of the 212 mothers, 36 (17.0%) did not swallow nevirapine doses before they gave birth. Of the 176 mothers who swallowed the nevirapine doses, 29 (16.5%) swallowed the tablets less than the recommended two hours before they delivered. This calculates to a maternal non-adherence rate of 65/212 (30.7%), taking into consideration the timing of the single-dose nevirapine tablet intake (Table 2).

**Table 2:** Prevalence of non-adherence to the maternal and newborn single doses of nevirapine in Bindura, 2008

Adherence Characteristics	Frequency		95%CI
	n = 212	%	
***Maternal nevirapine dose***			
Swallowed nevirapine at least 2 hrs before delivery	147	**69.3**	**62.9-75.3**
Swallowed nevirapine less than 2 hrs before delivery	29	**13.7**	**9.5-18.8**
Did not swallow nevirapine before delivery	36	**17.0**	**12.4-22.5**
Total maternal non-adherence to NVP	65	**30.7**	**24.7-37.1**
			
***Newborn nevirapine dose***			
Swallowed nevirapine within 72 hrs of birth	155	**73.1**	**66.8-78.8**
Swallowed the nevirapine more than 72 hrs after birth	15	**7.1**	**4.2-11.2**
Did not swallow nevirapine after birth	42	**19.8**	**14.9-25.6**
Total newborn baby non-adherence to nevirapine	57	**26.9**	**21.2-33.2**
			
***Combined maternal-infant nevirapine doses***			
Mother, baby or both took NVP within recommended time	121	**57.1**	**50.3-63.6**
Mother, baby or both took NVP outside recommended time	25	**11.8**	**8.0-16.7**
Mother, baby or both did not swallow nevirapine	66	**31.1**	**25.2-37.6**
Combined mother-baby non-adherence to NVP	91	**42.9**	**36.4-49.7**

### Infant adherence to nevirapine

Out of the 212 babies, 42 (19.8%) of them did not swallow nevirapine after birth. Of the 170 babies that swallowed the nevirapine, 15 (8.8%) took the dose after the recommended 72 hours of birth. This gives an infant nevirapine non-adherence rate of 57/212 (26.9%), after adjusting for the timing of the nevirapine intake (Table 2).

### Mother-baby pair adherence to nevirapine

Mother-baby pairs where either the mother or the baby or both failed to swallow nevirapine doses were 53 (25.0%), but when taking into consideration the recommended time frames of swallowing the nevirapine doses, 91 (42.9%) of the mother-baby pairs were non-adherent (Table 2).

### Nevirapine accessibility and maternal adherence

**Figure 1:**
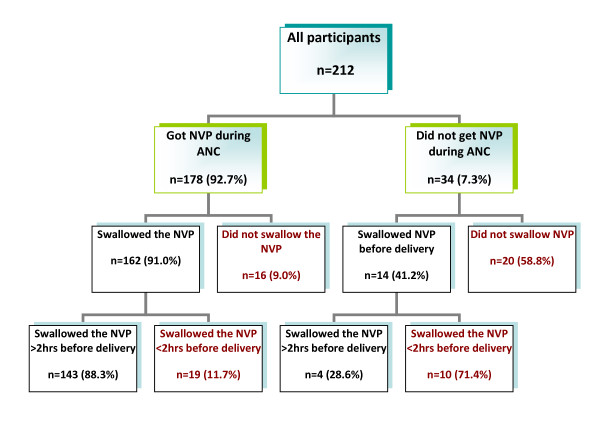
**The patterns of maternal nevirapine non-adherence in relation to the accessibity of the maternal dose of nevirapine, Bindura, 2008**. The flow diagram shows the adherence patterns of the mothers with reference to whether or not they received the nevirapine tablet during antenatal care (before the onset of labour).

Of the 212 mothers, 178 (84.0%) were given nevirapine tablets to take home during antenatal care (ANC). Among the 34 mothers who were not given nevirapine tablets during ANC, 20 (58.8%) did not ingest nevirapine before delivery and 14 (6.6%) got the nevirapine doses during labour. Of those who got the NVP tablet during labor, 71.4% ingested the doses less than 2 hrs before giving birth. (Figure 1)

Also of note was that among the 178 mothers who received the nevirapine tablets during ANC, 16 (9.0%) did not ingest the tablets before giving birth. Reasons given for not ingesting the NVP include forgetting to take tablet (4/16), misplacing the tablet (6/16), being away from home at onset of labour (4/16), labour progressed too fast (2/16) and that the church advised against taking tablet (1/16).

### Place of delivery and nevirapine adherence

Comparing adherence by place of delivery, 52.7% (48/91) of deliveries among the non-adherent group took place at home, compared to 5.0% (6/121) of deliveries among the adherent group. The reasons given for delivering at home (n = 54) include the unavailability of transport (42.6%), inability to afford paying for the transport (18.5%), clinic was closed (14.8%)], labour progressed too fast (7.4%)] and that the church discouraged them from going to the hospital (3.7%).

Among the home deliveries (n = 54), most (91.7%) of the non-adherence was of the infant NVP dose. Of the 42 infants that did not get nevirapine after birth, 76.2% were born at home. The reasons given for the babies not getting NVP included the clinic being too far (66.7%), mother not knowing that the baby was supposed to get NVP (11.9%), the baby was ill after birth (9.5%), the clinic was closed (4.8%) and husband refused (4.8%).

### Self-disclosure of HIV results and nevirapine adherence

More than half, 56.2%, of the mothers in the adherent group disclosed their HIV results to their partners compared to only 37.4% of the mothers in the non-adherent group. Furthermore, 73.6% of the mothers in the adherent group disclosed their HIV results to other people (who were not their partners), compared to 57.1% of the mothers in the adherent group.

### Ability to afford paying for ANC services and nevirapine adherence

The majority (93.9%) of the 212 mothers were asked to pay for antenatal services. Only 53.0% of the mothers in the non-adherent group indicated that they could afford paying the ANC charges, compared to 79.3% of the mothers in the adherent group.

### Factors associated with nevirapine non-adherence: bivariate analyses

Mother-baby pair non-adherence to nevirapine was associated with residing in a farming/rural settlement, inability to afford paying for ANC services, lack of maternal secondary education, staying with in-laws in the same household at the time of delivery, belonging to a religion not allowing the use of modern medicine, maternal use of traditional herbs during pregnancy, maternal non-disclosure of HIV results to partner, making less than three antenatal attendances, and giving birth at home. Previous maternal exposure to PMTCT and giving the mother a nevirapine tablet to take home during antenatal care were associated with reduced risk of non-adherence. We also looked at maternal employment status and multi-parity but these were not associated with mother-baby pair non-adherence on bivariate analysis. (Table 3).

**Table 3:** Factors associated with non-adherence to the nevirapine prophylaxis in Bindura: Bivariate analyses

Variable	SD-NVP Adherence status				POR	95% CI
	Non-Adherent		Adherent			
	n = 91	(%)	n = 121	(%)		
Resides in a farming or rural settlement	41	(45.1)	23	(19.0)	**3.49**	**1.89-6.45**
Unable to afford paying for health services	39	(42.9)	24	(19.8)	**3.40**	**1.82-6.33**
No maternal secondary education	42	(46.2)	20	(16.5)	**4.33**	**2.30-8.15**
Stays with in-laws in same household	20	(22.0)	14	(11.6)	**2.15**	**1.02-4.54**
Belongs to a restrictive religion	31	(34.1)	23	(19.0)	**2.20**	**1.17-4.12**
Used herbal medicine during pregnancy	29	(31.9)	12	(9.9)	**4.25**	**2.02-8.92**
Did not disclose HIV status to partner	57	(62.6)	53	(43.8)	**2.15**	**1.23-3.75**
Attended less than three ANC sessions	14	(15.4)	5	(4.1)	**4.22**	**1.46-12.19**
Delivered at home	48	(52.7)	6	(5.0)	**21.40**	**8.54-53.59**
Exposed to PMTCT in previous pregnancy	17	(18.7)	56	(46.3)	**0.27**	**0.14-0.50**
Received a nevirapine tablet during ANC	61	(67.0)	117	(96.7)	**0.07**	**0.02-0.20**
Mother not gainfully employed	68	(74.7)	80	(66.1)	1.16	0.84-3.10
Multi-parity (4^th ^child and above)	25	(27.5)	21	(17.4)	1.80	0.93-3.48

On stratified analysis, the mother's education level modified the association between the maternal employment status and non-adherence to NVP. Unemployed mothers who had attained secondary education were less associated with non-adherence (POR = 0.11, 95%CI: 0.01-0.87) than unemployed mothers who had not attained secondary education (POR = 3.36, 95%CI: 1.43-7.91). Distance from home to the nearest health facility also modified the association between the number of ANC sessions attended and non-adherence to nevirapine. Mothers who attended more than two ANC sessions and stayed within 5 km of a health facility were less likely to be non-adherent (POR = 0.07, 95%CI: 0.01-0.58) than those who attended more than two ANC sessions but resided more than 5 km from a health facility (POR = 1.09, 95%CI: 0.29-4.12).

### Factors associated with nevirapine non-adherence: logistic regression analyses

Lack of maternal secondary education (POR = 2.58, 95%CI: 1.11-6.00) and home deliveries (POR = 24.87, 95%CI: 8.37-73.93) were independently associated with mother-baby pair non-adherence to nevirapine, while previous maternal exposure to PMTCT (POR = 0.17, 95%CI: 0.07-0.44) and giving the mother a nevirapine tablet during antenatal care (POR = 0.06, 95%CI: 0.02-0.21) independently reduced the non-adherence. (Table 4)

**Table 4:** Factors associated with non-adherence to the SD-NVP regimen in Bindura: Logistic regression analyses

Risk Factor	POR	95% CI
**Combined mother-baby pair non-adherence to nevirapine**		
No maternal secondary education	**2.58**	**1.11-6.00**
Delivered at home (out of the health system)	**24.87**	**8.37-73.93**
Exposed to PMTCT in previous pregnancy (s)	**0.17**	**0.07-0.44**
Mother given a nevirapine tablet to take home during ANC	**0.06**	**0.02-0.21**
		
**Non-adherence to the maternal dose of nevirapine**		
No maternal secondary education	**2.38**	**1.05-3.39**
Multi-parity (4^th ^baby and above)	**2.66**	**1.05-6.72**
Mother given a nevirapine tablet to take home during ANC	**0.03**	**0.01-0.09**
Exposed to PMTCT in previous pregnancy (s)	**0.22**	**0.08-0.57**
		
**Non-adherence to the infant dose of nevirapine**		
Born at home (out of the health system)	**48.76**	**17.51-135.82**
Mother did not disclose HIV results to partner	**2.75**	**1.04-7.32**

Further logistic regression analyses were done after splitting the non-adherence into the maternal and infant SD-NVP components. Maternal non-adherence to NVP was independently associated with lack of maternal secondary education (POR = 2.38; 95%CI: 1.05-3.39) and multi-parity (POR = 2.66; 95%CI: 1.05-6.72) while previous maternal exposure to PMTCT (POR = 0.22; 95%CI: 0.08-0.57) and giving the mother a NVP tablet to take home during ANC (POR = 0.03; 95%CI: 0.01-0.09) independently reduced maternal non-adherence. Infant non-adherence to nevirapine was associated with maternal non-disclosure of HIV results to sexual partner (POR = 2.75; 95%CI: 1.04-7.32) home deliveries (POR = 48.76; 95%CI: 17.51-135.82). (Table 4)

### Availability of resources for PMTCT

Drugs for PMTCT prophylaxis (nevirapine tablets and nevirapine suspension) and rapid HIV testing kits were available in all the four participating health facilities throughout the study period. Each of the four health centres had at least two nurses trained on PMTCT working in the PMTCT sections, though there was a general shortages of nurses in the institutions.

## Discussion

Non-adherence to the SD-NVP regimen was high in Bindura, compared to some previous studies [11,14] though it was comparable to one Kenyan study where they found non-adherence rates of up-to 55% [15]. The high non-adherence in our study was probably because our definition of non-adherence included the ingestion of a NVP dose outside the recommended times. Some previous studies defined non-adherence only as the failure to ingest NVP [11,14]. Successful PMTCT prophylaxis also depends on the timing of the NVP ingestion. If the maternal NVP dose is ingested less than 2 hours before delivery the concentration in the blood may not reach enough therapeutic levels before giving birth and if the infant NVP dose is swallowed after 72 hours of birth the baby may not get the full benefits of the drug [16,17]. It is therefore important to consider the timing of the NVP intake when looking at adherence to the NVP regimen. While most previous studies looked at adherence only to the maternal NVP dose, our study defined non adherence looking at both the maternal and the infant doses of NVP. The infant dose of NVP is equally important for the regimen to be effective and thus the prophylaxis cannot be considered complete without administration of the infant NVP dose. A few studies have also looked at both maternal and infant adherence to PMTCT prophylaxis [11,18].

Of concern was that most babies born at home did not receive their NVP doses or received them too late. We attributed this to cultural practices in the studied population; newborn babies are confined within the house until the shedding of the umbilical cord stump, which usually takes about a week. The low self-disclosure of HIV results by the HIV positive mother also makes it difficult for her to convince the partner or the relatives of the need to take the baby to the clinic for the NVP dose before the week lapses. Other studies have also reported an association between home deliveries and babies missing their NVP doses [19,20].

Non-adherence to the maternal dose of NVP was more than twice higher among mothers who had no secondary education. Maternal education enhances communication between the mother and healthcare providers and also improves retention of provided information, leading to better implementation of recommended interventions. Education also empowers the woman to have autonomy in making important decisions without relying on other people. Similar findings have been reported in previous studies [11,19].

NVP non-adherence was four times higher among mothers who attended less than three antenatal sessions during pregnancy. Attending more ANC sessions offers more opportunities for healthcare providers to reinforce issues such as the importance of taking the NVP at the recommended times, the importance of delivering institutionally, the need to take the baby to the clinic in the event that birth takes place at home, and others which have a bearing on adherence to NVP prophylaxis. Investigators in a similar study in Rwanda also found an association between non-adherence to PMTCT protocols and attending few ANC sessions [19].

Maternal non-adherence to NVP was almost three times higher in multi-parous mothers. Such women usually experience precipitate labour pains which are often detected late, resulting in delayed ingestion of prophylactic doses. Similar findings were reported in Zambia [11].

Giving the mothers NVP tablets to take home during ANC improved adherence. The woman who gets NVP in advance can ingest the pill even if she gives birth at home. Also among the mothers who deliver in health institutions, those who get the NVP in advance are more likely to ingest the tablets on time than those who wait to take the NVP doses on arrival to the hospital labour ward.

There are some limitations in this study that are worth noting. Our measurement of non-adherence relied on accurate reporting by the mother and could have been affected by recall bias since some of the mothers delivered more than 6 weeks prior to the interviews, and their records did not indicate the timing of NVP ingestion. It is also possible that some women who failed to ingest their NVP doses could have falsely claimed otherwise, making the results to underestimate the prevalence of non-adherence. Lastly, this study only looked at mothers who reported for postnatal care, there is the possibility that those women who did not attend postnatal care could be more non-adherent, possibly underestimating the true non-adherence.

## Conclusions

Non-adherence to the SD-NVP regimen was high in Bindura. Giving the HIV positive mother a nevirapine dose to take home during antenatal care, giving birth in health institutions and self disclosure of HIV results by the mother to the partner were associated with reduced nevirapine non-adherence. Nurses working in the PMTCT clinics should ensure that HIV positive mothers are given nevirapine tablets at first contact, regardless of the stage of the pregnancy. Health promotion should be intensified at the community level to encourage ANC attendance and institutional deliveries. The government should consider adopting a policy of dispensing infant NVP doses during ANC, as is done for the maternal dose, and the possibility of incorporating traditional birth attendants into the PMTCT programme, so as to increase access to the ARV prophylaxis.

## Abbreviations

ANC: Antenatal Care; ARV: Antiretroviral; CDC: Centers for Disease Control and Prevention (in Atlanta); Epi Info: Epidemiological Information System; HIV: Human Immunodeficiency Virus; NVP: Nevirapine; PMD: Provincial Medical Director; PMTCT: Prevention of Mother-to-Child Transmission of HIV; POR: Prevalence Odds Ratio; SD-NVP: Single Dose Nevirapine.

## Competing interests

The authors declare that they have no competing interests.

## Authors' contributions

LRK participated in developing the study concept, designing the study, acquisition of data, statistical analysis and drafting the manuscript. CDT participated in the design of the study and the acquisition of data. GNS contributed to designing of the study, seeking ethical approval, analysis of data and the revision of the manuscript. MT was involved in design, analysis and interpretation of data. All authors read and approved the final manuscript.

## Pre-publication history

The pre-publication history for this paper can be accessed here:

http://www.biomedcentral.com/1471-2458/10/218/prepub
